# Corrigendum

**DOI:** 10.1111/jdi.13252

**Published:** 2020-05-18

The authors would like to draw the reader’s attention to the errors in the following article.

Haneda M, Noda M, Origasa H, Noto H, Yabe D, Fujita Y, Goto A, Kondo T, Araki E. Japanese Clinical Practice Guideline for Diabetes 2016. *J Diabetes Investig* 2018; 9: 657–697.

In Table 5 on page 677, ‘Present’ and ‘Absent’ under ‘Coronary artery disease’ column should have been in the order of ‘Absent’ and ‘Present’, as seen in the revised table below.

**Table 5:** The lipid control target values in patients with diabetes

Coronary artery disease	Lipid control target values (mg/dL)			
	LDL‐C	HDL‐C	TG	Non‐HDL‐C
Absent	<120	≥40	<150	<150
Present	<100			<130

The authors apologize for the error and any confusion it may have caused.

